# *Escherichia coli* β-Lactamases: What Really Matters

**DOI:** 10.3389/fmicb.2016.00417

**Published:** 2016-03-30

**Authors:** Priyanka Bajaj, Nambram S. Singh, Jugsharan S. Virdi

**Affiliations:** Microbial Pathogenicity Laboratory, Department of Microbiology, University of Delhi South CampusNew Delhi, India

**Keywords:** β-lactamase, commensal, detection, *E. coli*, genotypes, rapid diagnostics, resistance genes

## Abstract

*Escherichia coli* strains belonging to diverse pathotypes have increasingly been recognized as a major public health concern. The β-lactam antibiotics have been used successfully to treat infections caused by pathogenic *E. coli*. However, currently, the utility of β-lactams is being challenged severely by a large number of hydrolytic enzymes – the β-lactamases expressed by bacteria. The menace is further compounded by the highly flexible genome of *E. coli*, and propensity of resistance dissemination through horizontal gene transfer and clonal spread. Successful management of infections caused by such resistant strains requires an understanding of the diversity of β-lactamases, their unambiguous detection, and molecular mechanisms underlying their expression and spread with regard to the most relevant information about individual bacterial species. Thus, this review comprises first such effort in this direction for *E. coli*, a bacterial species known to be associated with production of diverse classes of β-lactamases. The review also highlights the role of commensal *E. coli* as a potential but under-estimated reservoir of β-lactamases-encoding genes.

## Introduction

Since its discovery in 1885, the status of *Escherichia coli* has evolved truly. While the commensal strains for the most part have been shown to lack specialized virulence determinants and be beneficial to their host, pathogenic *E. coli* have been reported to cause a spectrum of diseases (Kaper et al., 2004; Baker, 2015). Among the intestinal pathogenic *E. coli*, the enterotoxigenic *E. coli* (ETEC) and the enteropathogenic *E. coli* (EPEC) have been recognized as the most common cause of bacterial gastroenteritis especially, in low income countries with poor sanitation conditions (Qadri et al., 2005; Croxen et al., 2013). In the developed countries, however, diarrhea caused by *E. coli* is usually observed as cases of traveler’s diarrhea (caused by ETEC strains) or sporadic occurrences (Centers for Disease Control and Prevention, 2016). In these developed parts of the world, the enterohemmorhagic *E. coli* (EHEC) represent the most important group of intestinal pathogenic *E. coli*, and have been associated with severe morbidity and mortality (Riley et al., 1983; Majowicz et al., 2014). Distinct from the commensals and intestinal pathogens, the extraintestinal pathogenic *E. coli* (ExPEC) cause infections of the urinary tract, bloodstream, cerebrospinal fluid, respiratory tract, and peritoneum. The pathology in such infections may be observed as cholecystitis, bacteremia, cholangitis, urinary tract infection (UTI), or neonatal meningitis. Infections caused by the ExPEC strains have been widely reported in the community settings as well as hospitals and long-term care facilities thus, causing a pronounced burden on the medical and economic resources across the globe (Russo and Johnson, 2003; Riley, 2014; Poolman and Wacker, 2016).

Different options have been suggested for treating the infections caused by diverse pathogenic *E. coli*. For instance, *E. coli* diarrhea is usually considered self-limiting or treated by maintaining adequate rehydration. However, in most instances especially cases of life threatening extraintestinal infections, antibiotics form the mainstay of treatment. In this regard, β-lactam antibiotics have long been used successfully (Paterson and Bonomo, 2005; Pfeifer et al., 2010). However, concomitant with their introduction during the early 1980s, β-lactamases conferring resistance to third generation cephalosporins were documented in several studies (Jacoby et al., 1988; Jarlier et al., 1988). These primarily included the extended-spectrum β-lactamases (ESBLs) observed either as the variants of TEM-1, TEM-2, and SHV-1 (Philippon et al., 1989; Bradford, 2001), or the CTX-M enzymes (Bonnet, 2004). Additional resistance to cephamycins and carbapenems soon followed by the emergence of the AmpC β-lactamases (Philippon et al., 2002; Jacoby, 2009) and carbapenemases (Livermore, 2009; Kumarasamy et al., 2010; Nordmann et al., 2011a).

Extensive information is available on detection, distribution, classification and molecular characterization of β-lactamases (Bradford, 2001; Philippon et al., 2002; Queenan and Bush, 2007; Jacoby, 2009; Nordmann et al., 2011a). However, much of this information is being published from the perspective of the enzyme *per se*, i.e., ‘β-lactamases’, with much less attention to organism-specific details. A platform which succinctly provides such details for individual bacterial species having clinical relevance to humans is highly desirable. This would help the modern microbiologists and clinicians to develop targeted combat strategies and address better the public health concerns raised by antibiotic resistant pathogens. As a first effort of its kind in this direction, the present review focuses on what needs to be culled out from the vast knowledge on “β-lactamases mediating antibiotic resistance in *E. coli*” – what is more relevant today. In the latter part, this review also addresses the role of commensal *E. coli* as potential reservoir of β-lactamase-encoding genes.

## β-Lactamases of Critical Concern

### CTX-M Type ESBLs

Although, more than 150 different TEM- and SHV- type ESBLs are known (http://www.lahey.org/Studies/), the CTX-M enzymes have been recognized as the most prevalent among *Enterobacteriaceae* (Bush, 2010; Cantón et al., 2012; D’Andrea et al., 2013; Day et al., 2016). In fact, *E. coli* was the first species in which the CTX-M type ESBLs were reported as early as 1990 (Bauernfeind et al., 1990). Since then, the *E. coli* strains carrying CTX-M have been isolated from both nosocomial and community-acquired infections (Bonnet, 2004; Woodford et al., 2004; Smet et al., 2010). More recently, the CTX-M phenotype has also been reported in *E. coli* strains isolated from healthy humans, livestock, companion animals, food products, and sewage (Pallecchi et al., 2007; Ewers et al., 2012; Tacão et al., 2012; Zheng et al., 2012; Franz et al., 2015), indicating the large-scale of the reservoirs harboring and disseminating these ESBLs.

Such global dissemination of the CTX-M type ESBLs has primarily been attributed to the extremely mobilizable genetic platforms which harbor *bla*_CTX-M_ genes (Cantón et al., 2012), association of these platforms with *E. coli* genotypes showing clonal dissemination (Coque et al., 2008; Nicolas-Chanoine et al., 2008; Woodford et al., 2011), and frequent co-existence of *bla*_CTX-M_ with genes conferring resistance to other classes of antibiotics like fluoroquinolones and aminoglycosides leading to high rates of co-selection (Cantón and Ruiz-Garbajosa, 2011; Bajaj et al., 2015). Analysis of the genetic environments of *bla*_CTX-M_ genes has revealed that the promoter sequence present in the upstream region significantly affects gene expression as well as its dissemination (summarized in **Table 1**). The genetic elements downstream of the genes encoding CTX-M ESBLs, on the other hand, have been identified in most instances but very little has been established regarding their association with a specific type of *bla*_CTX-M_ or their role in gene selection and mobilization, if any.

**Table 1:** Details of the upstream regions associated with different *bla*_CTX-M_ genes.

Upstream region	*bla*_CTX-M_	Reference
IS*Ecp1*	Most *bla*_CTX-Ms_	Poirel et al., 2005; Cantón and Coque, 2006
Intact IS*Ecp1*or its remnants flanked by IS*26*, 48 bp upstream of CTX-M encoding gene	*bla*_CTX-M-15_ and most genes encoding for CTX-M-1 group	Cantón and Coque, 2006; Eckert et al., 2006
ORF513, often associated with complex *sul1*-type integron bearing a common region (CR1)	*bla*_CTX-M-9_	Sabaté et al., 2002; García et al., 2005; Novais et al., 2006
Phage related invertase	*bla*_CTX-M-10_	Oliver et al., 2005
IS*Ecp1* disrupted by IS*50*	*bla*_CTX-M-25_	Munday et al., 2004

In particular, the *E. coli* strains carrying CTX-M-14 and CTX-M-15 ESBLs have been observed notably for their widespread dissemination (Bonnet, 2004; Coque et al., 2008; Nicolas-Chanoine et al., 2008). These have also been reported to show differential demographic associations such that the strains producing CTX-M-15 ESBLs have been mainly isolated from patients related to the Indian subcontinent whereas, those producing the CTX-M-14 ESBLs have been isolated from patients related to China and the rest of the South East Asia (Hawkey and Jones, 2009; Freeman et al., 2012; Liao et al., 2015). The *E. coli* strains carrying CTX-M-14 or CTX-M-15 ESBL have also been observed for differential susceptibilities to fluoroquinolones and amoxicillin-clavulanic acid (Freeman et al., 2012). This infact, should be considered as an important observation because both these antimicrobials constitute important alternatives for the treatment of infections caused by β-lactamase-producing strains of *E. coli*.

Furthermore, it has been observed that unlike majority of CTX-Ms which exhibit better hydrolysis of cefotaxime (cefotaxime minimum inhibitory concentrations, MIC ≥ 64 μg/ml) than ceftazidime (ceftazidime MIC 2–8 μg/ml), CTX-M-15 possesses increased activity against both these antibiotic substrates (Poirel et al., 2001; Paterson and Bonomo, 2005). In most instances, the *E. coli* strains associated with the carriage of CTX-M-15 type ESBLs have been identified to belong to the international sequence type (ST) 131 (Coque et al., 2008; Nicolas-Chanoine et al., 2008; Peirano et al., 2014b); discussed in greater details with other major resistance-associated genotypes.

### AmpC β-Lactamases

The AmpC β-lactamases comprise another important group of β-lactamases from *E. coli* which exhibit a hydrolytic profile similar to the ESBLs while having an additional hydrolytic activity toward cephamycins like cefoxitin and cefotetan. The inhibition of AmpCs is usually caused by cloxacillin and boronic acid, but not by the common β-lactamase inhibitors viz. clavulanic acid and tazobactam (Bradford, 2001; Jacoby, 2009; Thomson, 2010; Helmy and Wasfi, 2014).

In *E. coli*, regulation of AmpC production by the chromosomal *ampC* gene is distinctly characterized by the lack of AmpR which constitutes a member of the LysR transcriptional regulator family (Honoré et al., 1986). Therefore, unlike most other members of the family *Enterobacteriaceae, E. coli* exhibits a non-inducible AmpC phenotype (Jaurin et al., 1981) and the wild-type strains constitutively produce low levels of AmpC enzyme. In most cases, hyperproduction is governed by mutations in the promoter/attenuator region that maps between –42 to +81 positions with respect to the *ampC* open reading frame (Caroff et al., 1999; Nelson and Elisha, 1999; Peter-Getzlaff et al., 2011; Haenni et al., 2014). In others, the chromosomal AmpC hyperproduction has been reported to be caused by the presence of >1 copy of the *ampC* gene, incorporation of a stronger promoter sequence as part of an insertion element, or acquisition of stronger promoter from other bacterial species (Jaurin et al., 1981; Jaurin and Normark, 1983). It is important to note that irrespective of the mechanism involved, all such strains are collectively known as ‘derepressed mutants’ (Hanson and Sanders, 1999).

Besides hyperperproduction of the chromosomally encoded enzyme, the presence of one or more plasmid-mediated *ampC* genes has often been observed as a common, rather more widespread mechanism of production of high levels of AmpC. Based on the sequence similarities, six families of plasmid-mediated AmpC β-lactamases were described by Pérez-Pérez and Hanson (2002) as CIT, FOX, MOX, DHA, EBC, and ACC. Among the strains of *E. coli*, the most commonly recognized plasmid-mediated AmpC includes the CMY-2 type which belongs to the CIT family and shares homology with the chromosomally encoded AmpC from *Citrobacter freundii* (Sidjabat et al., 2009; Oteo et al., 2010; Helmy and Wasfi, 2014). Single nucleotide variants of *bla*_CMY -2_ have recently been described and have been associated with extended-spectrum of activity and increased MICs of cefotaxime, ceftazidime, cefepime and aztreonam (Hentschke et al., 2011; Kotsakis et al., 2013). Another variant of CMY-2 namely, CMY-13 has been shown to be regulated by a functional AmpR and exhibit an inducible expression similar to the DHA enzymes but unlike the other CMY types (Miriagou et al., 2004). Although, naturally occurring variants have been described within other families of plasmid-mediated AmpC, the effects of mutations on enzyme activity were either not substantial or not studied much.

### Carbapenemases Including Metallo β-Lactamases

Since, ESBL and AmpC producing *E. coli* strains are being frequently reported worldwide, carbapenems possibly represent the last alternative for effective treatment of life threatening infections (Thomson, 2010; Nordmann et al., 2011a; Poirel et al., 2016). One of the most common mechanisms of carbapenem resistance is mediated by the production of carbapenemases which represent β-lactamases of the most diverse hydrolytic potential (Queenan and Bush, 2007).

Detailed biochemical and epidemiological characteristics of each of the carbapenemase classes *viz.* A, B, and D have been described in depth in the reviews by Queenan and Bush (2007) and Nordmann et al. (2011a). Herein, it is important to note that the most widespread and clinically significant representatives of each class namely *Klebsiella pneumoniae* carbapenemases (KPC, class A); metallo-β-lactamases (MBLs, class B); and oxacillin-hydrolyzing metallo-β-lactamases (OXA, class D) have frequently been reported in different *E. coli* strains (Urban et al., 2008; Kumarasamy et al., 2010; Beyrouthy et al., 2013). Despite this, it is also noteworthy that in most instances the production of KPC and OXA carbapenemases as well as MBLs of the IMP and VIM type has been associated with strains of *K. pneumoniae* and *Acinitobacter baumannii* (Afzal-Shah et al., 2001; Nordmann et al., 2009, 2011a; Pournaras et al., 2010; Bartolini et al., 2014).

Strains of *E. coli*, nevertheless have been notorious for the production of a metallo-β-lactamase known as the New Delhi metallo-β-lactamase (NDM-1) (Kumarasamy et al., 2010; Nordmann et al., 2011b). High prevalence of the *E. coli* carrying NDM-1 has been observed in the Indian subcontinent where the organism *per se* represents one of the most common causes of diarrheal and other community-acquired diseases. The resultant high possibility of environmental contamination and spread of the *bla*_NDM-1_ gene has been supported by the trans-continental spread of NDM-1 producing strains from India and Pakistan to the UK (Kumarasamy et al., 2010), Australia (Poirel et al., 2010), and US (Peirano et al., 2011). Frequent co-existence of the *bla*_NDM-1_ gene on plasmids carrying other resistance genetic elements (Moellering, 2010; Nordmann et al., 2011b) is also suggested to contribute to the selection and worldwide dissemination of multi-drug resistance phenotype, making *E. coli* strains carrying *bla*_NDM-1_ a global public health concern.

### Other Notable β-Lactamases

The category primarily includes inhibitor-resistant and complex mutant β-lactamase variants of the TEM and SHV types. The strains of *E. coli* have been most commonly associated with these variants (Robin et al., 2011), which have been seen to evolve by both *in vitro* and *in vivo* antibiotic selective pressures (Barlow and Hall, 2002; Jacquier et al., 2013). While several mechanisms of resistance to β-lactam-β-lactamase inhibitor combinations have been suggested in *E. coli*, production of inhibitor-resistant TEMs (IRTs) or OXA-1 enzymes has been reported to predominate as the mechanism conferring resistance to amoxicillin-clavulanic acid – a widely used antibiotic for the treatment of *E. coli* infections. Such amoxicillin-clavulanic acid resistant strains of *E. coli* have been observed to be more clonal, but with lesser content of virulence genes than the susceptible strains (Oteo et al., 2014). Similar, albeit not yet completely characterized mechanisms have been suggested to provide resistance to another commonly used antibiotic-inhibitor combination viz. ampicillin-sulbactam (Cantón et al., 2008).

Besides the IRTs, an S130G inhibitor-resistant mutation associated with the SHV enzyme in the *E. coli* clinical isolate was reported by Prinarakis et al. (1997) as early as. Since then, however, the *E. coli* strains carrying inhibitor-resistant SHV have not been very frequent in the clinics. In 2000, M. G. Page described another mutation at Y105 to confer resistance to clavulanic acid. However, no clinical strain harboring this mutation has been reported so far. The main reason behind the absence of Y105 mutation in clinical strains has been suggested as the significance of position 105 in both substrate and inhibitor interaction (Li et al., 2012). Thus, inhibitors targeting residue 105 may be protected because inhibitor-resistant mutation also tends to decrease the overall efficiency of enzyme catalysis. This position has therefore, been suggested to be an important target for the design of newer inhibitors of broad-spectrum β-lactamases. Other positions in the SHV enzymes viz. N276, K234, and R244 have also been shown to confer reduced susceptibility to β-lactam-clavulanic acid combinations (Thomson et al., 2007; Manageiro et al., 2010; Winkler et al., 2015). The position R244, however, has interestingly been shown to have differential effects on the different β-lactamase inhibitors – clavulanic acid and sulbactam (Thomson et al., 2007).

It is noteworthy that the CTX-M type ESBL variants showing reduced susceptibilities to β-lactam-β-lactamase inhibitor combinations have not yet been observed. This could most likely be attributed to the limited exposure of mutant strains to such combinations or rather the recommended use of carbapenems as the treatment of choice (Pitout, 2010; Ortega et al., 2012); or limitations in detection procedures leading to confusions with other mechanisms such as the expression of IRTs or complex mutant TEMs (CMTs) (Ripoll et al., 2014). Nevertheless, an increase in the use of antibiotic-inhibitor combinations in the clinical settings is quite well-expected to result in the emergence and dissemination of strains carrying the inhibitor-resistant CTX-Ms. Infact, experiments using a collection of hypermutagenic *E. coli* strain GB20 carrying different *bla*_CTX-M_ genes have shown that such possibilities may be higher for the variants belonging to the CTX-M-1 group (Ripoll et al., 2011). Not to forget that the latter is already associated with the most widespread variants of the CMX-M ESBLs.

## Detection of β-Lactamases

### Phenotypic Detection

#### (a) Conventional Laboratory Methodologies

The standardized methodologies for phenotypic antibiotic susceptibility testing and breakpoints for interpretation of results are available primarily from Clinical and Laboratories Standards Institute (CLSI) and European Committee on Antimicrobial Susceptibility Testing (EUCAST). Although, both suggest a similar basic protocol for preliminary testing, variations are often observed in the suggested breakpoints (Clinical and Laboratory Standards Institute [CLSI], 2014a,b,c Document M02-A11, M07-A9, M100-24; European Committee on Antimicrobial Susceptibility Testing [EUCAST], 2014 Version 4.0).

For detection of ESBL-producing *E. coli*, both CLSI and EUCAST recommend the use of cefotaxime as well as ceftazidime as indicator cephalosporins, and employ *E. coli* ATCC 25922 as the wild type quality control strain. Besides this, the ESBL Etest available from bioMérieux (Marcy l’Etoile, France) or Cambridge Diagnostics Services Ltd (Cambridge, UK) is often used in many clinical microbiology laboratories.

Unlike ESBLs, no standardized guidelines are available from CLSI for the detection of AmpC producing *E. coli*. Nevertheless, in routine microbiology, a high MIC of cefoxitin (>8 mg/L) is considered to be an indicator of AmpC phenotype. Further confirmation by one or more methods is usually required and employs AmpC inhibitors like boronic acid derivatives or cloxacillin in a test format similar to the CLSI ESBL phenotypic confirmatory test (Brenwald et al., 2005; Tan et al., 2008; Tenover et al., 2009). Overall, the disk-based methods have been shown to be more sensitive and specific than the agar dilution methods and cefoxitin–cloxacillin combination disk method has been shown to provide the most accurate results (Tan et al., 2008).

Thus, like other *Enterobacteraceae*, it is easy to detect the *E. coli* strains which produce AmpC β-lactamases. However, differentiation of the chromosomal AmpC hyperproduction from the plasmid-mediated enzymes is not possible solely on the basis of phenotypic tests. Moreover, detection of inducible DHA-like AmpC β-lactamases requires the use of an additional test known as the ‘disk approximation assay’ wherein, an inducer and reporter antibiotic substrate are used and results are considered positive if the inhibition zone reduces by ≥2 mm on the induced side of the reporter disk (Dunne and Hardin, 2005).

Similar to the detection of AmpC β-lactamases, the guidelines for initial screening of carbapenemase-producing *E. coli* may be applied to other members of *Enterobacteriaceae* as well. Preliminary detection of carpapenemase production involves MIC breakpoints for imipenem, ertapenem and meropenem (Clinical and Laboratory Standards Institute [CLSI], 2014c, Document M100-24; European Committee on Antimicrobial Susceptibility Testing [EUCAST], 2014 Version 4.0). For confirmation of carbapenemase production, CLSI recommends the Modified Hodge test (MHT) (M100-S20). However, despite being a useful method for initial detection of carbapenemase production (especially, the KPC type), MHT has gained limited popularity because of the long time consumption, *ca.* 24–48 h; low specificity, in case of high ESBL and/or AmpC activity; and low sensitivity, mainly for the NDM-1 producers (Miriagou et al., 2010; Lutgring and Limbago, 2016).

For better detection of the diverse carbapenemase classes including, metallo-β-lactamases, different inhibitor-based methods have been suggested which often employ the algorithm proposed by Giske et al. (2011). These methods also allow differentiation of carbapenem resistance caused by the production of carbapenemases *per se* from that caused by a high AmpC activity, usually by incorporating cloxacillin in the plating medium (Giske et al., 2011; Nordmann et al., 2012a). However, lack of suitable inhibitors limits the application of inhibitor-based methods in the detection of OXA-48-like enzymes where, a high MIC of temocillin (>32 mg/L) or resistance to piperacillin-tazobactam with no AmpC production has been shown to provide good results (Glupczynski et al., 2012). Besides these, detection of NDM-1 producing *E. coli* can be performed by using Etest MBL strips (AB bioMérieux, Solna, Sweden) consisting of a gradient of imipenem concentrations on one side and imipenem with ethylenediaminetetraacetic acid (EDTA) on the other; or combined disk test using imipenem disks with and without EDTA (Franklin et al., 2006).

#### (b) Automated and Rapid Detection

The commercial methods available for rapid detection of ESBL producers include – VITEK 2 ESBL test (bioMérieux, Marcy l’Etoile, France), Phoenix ESBL test (Becton Dickinson, Sparks, MD, USA), and Microscan WalkAway-96 System (Dade Behring, West Sacramento, CA, USA). Besides these, MALDI-TOF MS or the recently described chromogenic and β LACTA tests have been suggested for rapid detection of *E. coli* strains producing ESBLs (Compain et al., 2015; Dortet et al., 2015).

Despite an overall similar performance of these automated systems, the results should be examined critically due to the possibility of upto 5% error based on antibiotic-organism combination and instrument handling. In case of ESBL-producing *E. coli* strains, a comparative analysis of the automated methods, double-disk synergy test, and combination disk methods has shown combination disk method as the best choice followed by ESBL Etest (Wiegand et al., 2007). However, it is important to consider that the accuracy of combination disk method is subject to inaccuracy when the strain under consideration co-produces AmpC β-lactamases (Drieux et al., 2008; Munier et al., 2010). Therefore, it is recommended that confirmation of ESBL production should be done by one or more additional tests. Some of these are suggested by the EUCAST guidelines for detection of resistance mechanisms and specific resistances of clinical and/or epidemiological importance (December 2012), and include the use of cefepime as an indicator cephalosporin in either of the test formats or the use of cloxacillin to inhibit AmpC β-lactamase activity (Drieux et al., 2008; Nourrisson et al., 2015).

The most commonly available commercial methods for rapid detection of AmpC β-lactamases include the AmpC Etest (bioMérieux, Marcy l’Etoile, France) and the D69C AmpC Detection Disc Set (Mast Group Ltd, UK). The D69C AmpC Detection Disc Set has been shown as a promising candidate for the detection of both, plasmid-borne as well as chromosomal AmpC, irrespective of their constitutive, inducible or derepressed nature (Halstead et al., 2012). More recently, Hart et al. (2015) have shown the detection of CMY-2 type AmpC β-lactamases among others in *E. coli* strains using MALDI-TOF MS analysis following in solution trypsin digestion of periplasmic proteins and nano-LC based separation.

Rapid methods to detect carbapenemases includes the Carba NP test (Nordmann et al., 2012b) or MALDI-TOF analysis of carbapenem hydrolysis (Hrabák et al., 2012; Lasserre et al., 2015). Furthermore, screening for carbapenemase producing *E. coli* strains among carriers can be performed by directly using stools or rectal swabs, or by following an overnight enrichment step in broth medium containing one of the carbapenems (i.e., imipenem – 0.5–1 mg/L or ertapenem – 0.5 mg/L) (Adler et al., 2011). In either case, different selective media may be employed with varying sensitivity and specificity. The most popular ones includes ChromID ESBL (bioMérieux, La Balme-les-Grottes, France) and CHROMagar KPC (CHROMagar Company, Paris, France). ChromID ESBL has been shown to provide high sensitivity but, it is often limiting in specificity especially, because of ESBL producers. CHROMagar KPC, on the other hand, has been shown to be more specific for carbapenemase producers but, exhibits lower sensitivity (Carrër et al., 2010). More recently, another medium *viz.* chromID CARBA (bioMérieux) has been shown to be an accurate method for detection of Enterobacterial strains which produce carbapenemases of KPC or NDM-1 types (Vrioni et al., 2012).

### Detection of β-Lactamase Encoding Genes

#### (a) PCR and Sequencing

Despite the availability of various phenotypic methods, molecular biology techniques (especially, PCR and sequencing) serve as the gold standard for detection, identification and differentiation of different β-lactamases (Bradford, 2001). Molecular methods have also been successfully applied for the detection and analysis of resistance which is often associated with low level of β-lactamase expression (e.g., that caused by the OXA-48 carbapenemases), and for the identification of the exact mechanism of resistance (e.g., differentiation of carbapenem resistance caused by a high ESBL/AmpC activity coupled with porin mutation from that caused by the production of carbapenemases *per se*) (Gazin et al., 2012). Further improvements in the speed, sensitivity, and specificity have been achieved by the establishment of multiplex and real time PCR assays (Pérez-Pérez and Hanson, 2002; Birkett et al., 2007; Brolund et al., 2010; Poirel et al., 2011; Deccache et al., 2015). For instance, a multiplex PCR has been successfully applied for the detection of different families of plasmid-mediated AmpC β-lactamases, and their differentiation from the chromosomally encoded enzyme (Pérez-Pérez and Hanson, 2002). Similarly, a real-time multiplex PCR has recently been proposed and evaluated for the detection of genes encoding different carbapenemases among Gram-negative bacteria (van der Zee et al., 2014).

Sequencing of the PCR amplicons helps not only in the identification of genes but, also in the detection of newer genetic variants within a particular β-lactamase class which often differ only by a single nucleotide. The technique is therefore important for recognition of nucleotide positions significantly associated with enzyme activity, determination of the epidemiology of resistance genetic elements, and tracing the emergence and dissemination of newer variants. Thus, it has been observed that *bla*_CTX-M-15_ and *bla*_CMY -2_, respectively, constitute globally the most disseminated genes for ESBL and plasmid-mediated AmpC produced by the *E. coli* strains (Nicolas-Chanoine et al., 2008; Oteo et al., 2010; Cantón et al., 2012; Helmy and Wasfi, 2014).

#### (b) High-Throughput Analyses

For high-throughput screening, different DNA microarrays and enzyme-linked immunosorbent assays (ELISA) have been developed. However, DNA microarrays have overtaken considerably as the method of choice and various advancements have been achieved over the recent years. The leading commercial DNA microarrays comprise the Check-Points assays (Check-Points Health, Wageningen, The Netherlands) and the Identibac AMR-ve assays (Alere GmbH, Cologne, Germany). While both possess a similar turnaround time and classify the CTX-M type ESBLs further into their respective groups, the latter exhibits lower specificity and does not differentiate between the genes encoding narrow-spectrum β-lactamases and ESBLs (Gazin et al., 2012).

Given the diversity of β-lactamase encoding genes in *E. coli*, the latest of the Check-Points assays called the ‘Check-MDR CT103 array’ appears as a promising platform for rapid screening as it has been shown to exhibit 100% sensitivity and specificity of detection for the major gene-families encoding ESBLs (*bla*_TEM_, *bla*_SHV_, and *bla*_CTX-M_), plasmid-mediated AmpCs (*bla*_CMY/MOX_, *bla*_DHA_, *bla*_FOX_, *bla*_ACC_, and *bla*_ACT/MIR_), and carbapenemases (*bla*_KPC_, *bla*_NDM_, *bla*_V IM_, and *bla*_IMP_). Additionally, the sensitivity and specificity for *bla*_OXA_-48 were 95 and 100%, respectively, (Cuzon et al., 2012).

Although, PCR and microarray based methodologies have been successfully applied in the detection and analysis of target genes encoding antibiotic resistance, these may not be sufficient to enable the discovery of rare or novel mechanisms of resistance. Whole genome sequencing has been increasingly advocated as a highly sensitive and specific approach in this regard. It has been shown to be especially useful for organisms having a complex pattern of antibiotic resistance; a classic example of which may be represented by *E. coli*. Several bioinformatics tools and databases (like ResFinder, ARG-ANNOT along with ClustalW, BLASTn, and/or BLASTx) have been developed to analyze the data obtained by whole genome sequencing and extract the antibiotic resistance genotypes including chromosomal mutations associated with resistance (Stoesser et al., 2013; Zankari et al., 2013). With appropriate cost reduction and methodological refinements whole genome sequencing seems sure to take over as a method of choice for resistance prediction in the near future.

## Resistance, Genotypes, and Global Dissemination

Several recent studies have described that the nature of antibiotic resistance in *E. coli* is often associated with the strains’ genotype. The most predominant of the multi-drug resistant (MDR) genotypes has been observed as the sequence type 131 (ST131) which belongs to phylogroup B2 and harbors extra-intestinal pathogenic strains mostly associated with urinary tract and bloodstream infections (Johnson et al., 2010; Courpon-Claudinon et al., 2011; Peirano et al., 2011; Rogers et al., 2011). The strains belonging to ST131 have been reported to carry several virulence genes which encode the aerobactin receptor (*iutA*), catecholate sidephore receptor (*iroN*), invasion of brain endothelium (*ibeA*), pathogenicity island marker (*malX*), secreted autotransporter toxin (*sat*), uropathogenic-specific protein (*usp*), and *fimH* adhesin of type 1 fimbriae (Rogers et al., 2011; Blanco et al., 2013).

The most characteristic feature of the *E. coli* ST131 isolates *vis-à-vis* β-lactamase mediated antibiotic resistance has been their association with the pandemic spread of resistance mediated by the CTX-M-15 type ESBL (Coque et al., 2008; Nicolas-Chanoine et al., 2008). This spread has been driven primarily by the strains belonging to a well defined clade within the subclone *H*30 which has been designated as *H30*-Rx owing to its broader resistance profile (Price et al., 2013; Peirano et al., 2014b). The major vehicles for pandemic spread of CTX-M-15 ESBL by *E. coli* strains belonging to ST131 have been recognized as plasmids especially, of the incompatibility groups IncF (e.g., pEK516, pEK499, pGUE-NDM, pC15-1a, pJJ1886-5, pEC_B24, pEC_L46, pEC_L8, pJIE186-2). Besides IncF, other plasmid incompatibility groups like IncK, IncX, and IncI have also been observed frequently in both ST131 and non-ST131 *E. coli* strains carrying CTX-M-15 ESBLs (Carattoli, 2013; Phan et al., 2015).

The ST131 strains have also been significantly associated with other CTX-M types as well as resistance to carbapenems, and other antibiotic classes like fluoroquinolones, trimethoprim-sulfamethoxazole, and aminolglycosides (Oteo et al., 2009; Peirano et al., 2011, 2014b; Rogers et al., 2011; Morris et al., 2012). However, their association with plasmid-mediated AmpC has been less-well established. On the contrary, the spread of AmpC-mediated antibiotic resistance (especially, the most widespread CMY-2 type) has been shown to be driven primarily by the strains belonging to phylogroup D, and has been mostly associated with ST38 and ST448 (Sidjabat et al., 2009; Naseer et al., 2010; Oteo et al., 2010; Matsumura et al., 2013). Nevertheless, acquisition and spread of plasmid-mediated *ampC* genes by ST131, even to a lesser extent poses a serious public health concern for near future because the clone enjoys a global distribution and has successfully driven the dissemination of *bla*_CTX-M-15_ worldwide.

Apart from the AmpC β-lactamases, contribution of *E. coli* strains from phylogroup D in the spread of genes encoding CTX-M type ESBLs has also been noteworthy (Coque et al., 2008; van der Bij et al., 2011; Hansen et al., 2014). In this regard, ST405 has been observed as the most predominant genotype which stands second only to the ST131 clones from phylogroup B2 (Coque et al., 2008). This sequence type has primarily been associated with the large scale dissemination of CTX-M-15 and CTX-M-9 group of ESBLs (Naseer and Sundsfjord, 2011). Other sequence types reported in association with CTX-M mediated antibiotic resistance include ST59, ST393, ST1395, and ST354 (CTX-M-14), ST38 (CTX-M-9), and ST648 (CTX-M-15) (Mora et al., 2011; van der Bij et al., 2011). More recently, the globally distributed multidrug resistant genotypes (especially, ST69 and ST393/O15:K52:H1) which have chiefly been known for resistance to antibiotic classes like trimethoprim-sulfamethoxazole have also been shown to be associated with resistance caused by the CTX-M type ESBLs (Johnson et al., 2009; Ewers et al., 2012; Izdebski et al., 2013; Hansen et al., 2014).

Thus, a major proportion of *E. coli* strains expressing ESBLs and plasmid-mediated AmpCs have been reported from the phylogroups (i.e., B2 and D) which have also been associated with higher virulence. Occasionally, however, similar resistance is also being reported from the sequence types belonging to phylogroups A and B1 (Oteo et al., 2009, 2010; Valverde et al., 2009). For instance, in a recent study, the predominant CTX-M-15-producing *E. coli* in the fecal carriage of travelers and immigrants were reported to belong to phylogroup A (ST10) (Valverde et al., 2015). Besides this, ST410 and ST224 from phylogroup A and B1, respectively, have also been associated with *E. coli* strains producing CTX-M-15 ESBL (López-Cerero et al., 2011; Mshana et al., 2011; Silva et al., 2016). Corvec et al. (2007) have also shown the existence of a significantly high association of hyperproduction of the chromosomally encoded AmpC with *E. coli* strains belonging to phylogroup A. However, despite these reports, the contribution of phylogroups A or B1 in the global epidemiology of β-lactamase mediated antibiotic resistance remains to be established more clearly because none of the sequence types reported so far have been shown to play a role in the worldwide dissemination of either ESBLs or AmpCs.

Last but not the least, relatively lesser information is available regarding genotypic or sequence type associations of carbapenemase-mediated resistance in *E. coli*. Some of the studies, as described above, have indicated the role of ST131 in the clonal spread of carbapenemases (Peirano et al., 2011, 2014a; Morris et al., 2012). Meanwhile, others have highlighted the importance and probably a greater role played by ST101 (phylogroup B1) and ST405 (phylogroup D) especially, in conjunction with the NDM-1 type enzymes (Mushtaq et al., 2011; Pfeifer et al., 2011).

## β-Lactamases and the Commensal *E. coli*

For better management of emergence and dispersal of resistance, complete analysis of the abundance, diversity, and dissemination of resistance genes in bacteria including commensals is required. One of the most significant efforts in this regard has been made by the Alliance for the Prudent Use of Antibiotics (APUA). Through its Reservoirs of Antibiotic Resistance (ROAR) project, APUA aims to encourage research for determining antibiotic resistance in commensals. It also works to provide a comprehensive database for further analyses like prediction of the rates of resistance development in pathogens by evaluating the frequency of resistance genes in commensals.

Through several such individual and collaborative efforts, it has been acknowledged that the association of commensal *E. coli* with genes imparting antibiotic resistance constitutes a serious public health concern especially, with respect to resistance dissemination through community settings (Valverde et al., 2008; Machado et al., 2013). Despite being a minor proportion of microbiota colonizing the gastrointestinal tract of healthy individuals, the role of commensal *E. coli* as a reservoir of genes encoding β-lactamases has been acknowledged across the globe (**Table 2**). Among these, a large number of studies have reported a high prevalence of CTX-M type ESBLs in the strains isolated from healthy adults and children (Nys et al., 2004; Pallecchi et al., 2007; Woerther et al., 2013; Nakayama et al., 2015). A number of studies have also demonstrated a likewise prevalence in food and companion animals (Ben Sallem et al., 2012; Tian et al., 2012; Zheng et al., 2012).

**Table 2:** World-wide occurrence of β-lactamase genes in commensal *E. coli.*

Countries reported from	β-lactamase genes identified	Reference
Kenya, Mexico, Peru, Philippines, Curacao, Venezuela, Ghana, Zimbabwe	ND	Nys et al., 2004
Latin America	*bla*_SHV,CTX-M_	Pallecchi et al., 2007
Japan	*bla*_CMY_	Kaneko et al., 2006
USA	*bla*_TEM, CTX-M_	Sommer et al., 2009
Sweden	*bla*_TEM, SHV, CTX-M_	Tängdén et al., 2010
Australia	*bla*_TEM_	Bailey et al., 2010
China	*bla*_TEM, SHV, CTX-M, CMY,KPC_	Tian et al., 2012
China	*bla*_CTX-M_	Zheng et al., 2012
Tunis	*bla*_TEM, CTX-M, CMY_	Ben Sallem et al., 2012
Thailand	*bla*_CTX-M_	Luvsansharav et al., 2012
Switzerland	*bla*_TEM, CTX-M_	Geser et al., 2012
Portugal	*bla*_TEM, CTX-M_	Machado et al., 2013
Netherlands	*bla*_TEM, SHV, CTX-M, CMY_	Hordijk et al., 2013
India	*bla*_TEM, SHV, CTX-M, CMY_	Kothari et al., 2013
India	*bla*_CTX-M_	Dureja et al., 2014
Germany	*bla*_TEM, SHV, CTX-M_	Valenza et al., 2014
China	*bla*_TEM, OXA_	Li et al., 2014
Nigeria	*bla*_CTX-M_	Fortinia et al., 2015
USA	*bla*_CTX-M,CMY_	Davis et al., 2015
Vietnam	*bla*_CTX-M_	Bui et al., 2015

*ND: Not Determined.*

Some of these studies have also indicated that animals might be responsible for the transfer of ESBL-producing bacteria and/or ESBL-encoding genes to humans, either through contact or *via* food chain (Jensen et al., 2006; Ewers et al., 2012). The indiscriminate use of antibiotics in the food and farming industry has therefore, been considered as one of the most significant risk factors responsible for high prevalence of ESBL producing *E. coli* among healthy subjects (Bailar and Travers, 2002; Larson, 2007). Antibiotic abuse related to consumption without prescription, and exposures related to family/community/foreign travel have been observed as other notable factors (Rodríguez-Baño et al., 2008; Tängdén et al., 2010; Luvsansharav et al., 2011).

Besides ESBLs, these factors have also contributed to the increased occurrence of AmpC β-lactamases in strains isolated from healthy humans and animals (Kaneko et al., 2006; Hammerum et al., 2011; Ben Sallem et al., 2012; Ewers et al., 2012). Occurrence of carbapenemase-producing *E. coli* in the community, however, has been reported in only a few studies. Furthermore, their acquisition by healthy humans has been associated chiefly with travel to the countries endemic in this regard (Tian et al., 2012; Ruppé et al., 2014).

Overall, more studies are required to assess the contribution of commensal *E. coli* strains in the epidemiology of genes encoding β-lactamases, and to understand the mechanisms by which these resistance genes are silently fed to the pathogenic strains.

## Other Important Considerations

Owing to the basic biology of the organism, the menace of ‘β-lactamase mediated antibiotic resistance in *E. coli*’ represents a concern which needs special attention. The genome of *E. coli* is known for its plasticity and thus, the ability to evolve constantly. Such genome plasticity enables the strains to survive under different environmental conditions and selective pressures (Dragosits et al., 2013; Laehnemann et al., 2014). Evolution under such conditions may be mediated by mutations, horizontal gene transfer and/or transposable elements. Thus, resistance to a particular antibiotic may be acquired through sequential mutations in the chromosomal DNA or by mobile genomic elements. In the latter case, the exchange of genetic material among bacteria leads to further transmission of resistance (Levy and Marshall, 2004; Barlow, 2009).

In *E. coli*, β-lactamase mediated resistance is dominated mainly by the acquisition and dissemination of genomic elements through horizontal gene transfer *viz.* conjugation, transformation and transduction (Doi et al., 2012; Huddleston, 2014). In this regard, it is noteworthy that pathogenic as well as commensal strains of *E. coli* occupy niches which provide ambient conditions for such gene transfer. In the host environments, the population of *E. coli* is infact more abundant than most other members of *Enterobacteriaceae* and thus, represents a predominant vehicle for transmission of resistance (O’Brien, 2002; Huddleston, 2014). Even in the natural environments such as urban wastewaters, *E. coli* strains are surrounded by conditions favorable for exchange of resistance-associated genomic elements, and evolution of newer resistance mechanisms especially in response to sub-inhibitory concentrations of antibiotics (Rizzo et al., 2013; Wellington et al., 2013). Furthermore, and as discussed in earlier sections, the compounding contribution of the clonal spread of β-lactamase mediated antibiotic resistance has been most noticeable in the species *E. coli*. The factors contributing to such clonal dissemination, however, need to be investigated in detail.

In view of the diverse and highly efficient means of resistance accumulation and dissemination in *E. coli*, suitable strategies for tackling drug resistance in this organism are highly warranted. The first step which may be advocated in this direction is the prudent use of currently available antibiotics which in turn requires rapid diagnostics to study susceptibilities in an organism-specific manner. Further, novel antibiotics which attack new target sites or block/circumvent existing resistance mechanisms need to be developed. To accomplish this, knowledge of the detailed mechanisms underlying resistance to particular class of antibiotic in the target organism represents an utmost necessity. The complex nature of mechanisms underlying β-lactamase mediated resistance in *E. coli* thus, represents a classic example which highlights the need for more such cohesive platforms providing information for other antibiotic-bacteria combinations.

## Concluding Remarks

The potential of *E. coli* to cause varied infections coupled with having several antibiotic resistance mechanisms is a real challenge to disease management strategies. Of particular concern is the resistance mediated by the newer β-lactamases since, third-generation cephalosporins and other higher generation β-lactam antibiotics have long served as the major mainstay for successful treatment of infections caused by *E. coli*. This review is an attempt to collate key information pertaining to β-lactamases of significance to *E. coli*. It also highlights the areas of work which needs to be explored in future especially, development of rapid diagnostics and understanding association of genotypes with specific resistance types. Studies should also be undertaken to investigate the role of commensal *E. coli* in the global epidemiology of β-lactamase mediated resistance. The authors suggest that such reviews specifically targeting clinically important pathogens or groups of pathogens would not only serve the clinicians engaged in the management of infections caused by resistant organisms but also provide valuable resource to those interested in designing organism-specific strategies for surveillance and control of resistance in the larger interest of public health.

## Author Contributions

PB reviewed the literature and wrote the manuscript. NS contributed in writing parts of the manuscript. JV conceived the study and checked the manuscript.

## Conflict of Interest Statement

The authors declare that the research was conducted in the absence of any commercial or financial relationships that could be construed as a potential conflict of interest.
